# Combined early palliative care for non-small-cell lung cancer patients: a randomized controlled trial in Chongqing, China

**DOI:** 10.3389/fonc.2023.1184961

**Published:** 2023-09-14

**Authors:** Mengting Chen, Huiqing Yu, Liejun Yang, Hong Yang, Haoyang Cao, Lei Lei, Liling Ma, Shihong Liu, Ling Tian, Sixiong Wang

**Affiliations:** ^1^ Department of Clinical Nutrition, Chongqing University Cancer Hospital, School of Medicine, Chongqing University, Chongqing, China; ^2^ Department of Geriatric Oncology and Department of Palliative Care, Chongqing University Cancer Hospital, School of Medicine, Chongqing University, Chongqing, China

**Keywords:** combined early palliative care, non-small-cell lung cancer (NLSCLC), overall survival (OS), pain management (MeSH), psychological status, nutritional level

## Abstract

**Purpose:**

More effective approaches are needed to improve the prognosis of non-small-cell lung cancer (NSCLC) patients. Thus, we used the E-warm model to assess how early integration of interdisciplinary palliative care was related to the quality of life (QoL), psychological functioning, pain management, and nutrition factors of NSCLC patients.

**Methods:**

This randomized controlled trial enrolled 280 newly diagnosed NSCLC patients, which were randomly divided (1:1) into combined early palliative care (CEPC) and standard oncological care (SC) groups. At baseline and after 24 weeks, the Functional Assessment of Cancer Therapy-Lung (FACT-L) scale, Hospital Anxiety and Depression Scale (HADS), and the Patient Health Questionnaire-9 (PHQ-9) were used to assess QoL and psychological function, respectively. The Numerical Rating Scale (NRS) and Patient-Generated Subjective Global Assessment (PG-SGA) were used to assess cancer patients’ pain and nutrition levels. The primary outcome was overall survival (OS). Secondary outcomes comprised changes in the QoL, psychological functioning, pain, and nutrition state. The intention-to-treat method was applied for analysis. This study was registered at www.chictr.org.cn (ChiCTR2200062617).

**Results:**

Of the 140 patients enrolled in the CEPC and SC groups, 102 and 82 completed the research. The CEPC group presented higher QoL than the SC group (*p* < 0.05). Additionally, fewer patients presented depressive symptoms in the CEPC group than in the SC group (*p* < 0.05), as well as better nutritional status (*p* = 0.007) and pain management (*p* = 0.003). Compared to the SC group, CEPC patients had significantly longer OS (20.4 *vs*. 24.6 months, *p* = 0.042; HR: 0.19; 95% CI: 0.04-0.85, *p* = 0.029).

**Conclusion:**

With combined early palliative care, NSCLC patients lived longer, had better QoL, were psychologically stable, were in less pain, and were more nutritionally satisfied.

## Introduction

About 85% of lung cancers are non-small-cell lung cancers (NSCLC), which leads to a poor quality of life (QoL) and high symptom burdens (1–4). It is common for NSCLC patients to be diagnosed at an advanced stage and, therefore, lose the opportunity to undergo radical resection (5–7). Moreover, NSCLC patients are often malnourished, in pain, and psychologically distressed, contributing to poor QoL and short survival (8, 9). Thus, NSCLC patients need high-quality, low-medical-burden interventions to improve their QoL, nutrition, psychological well-being, and survival.

Palliative care focused on managing symptoms and providing psychosocial support can improve a patient’s quality of life and care (10, 11). For advanced cancer patients, palliative care has accumulated substantial evidence supporting its integration into oncology practice (12–15). There has been a rapid expansion in palliative care services worldwide, but China is experiencing a fundamentally different situation. Several factors contribute to the problem, including limited healthcare resources, policies, low awareness, and local cultural norms (16–19). Palliative care in China is still in its infancy (20, 21). In the greater China region, models of palliative care that delivered specialist palliative care services in various settings were reported (21), including hospitals (22), inpatient hospice units (23, 24), nursing home (25), and home-based care (26–28). Furthermore, few studies have focused on the collaborative experience of various specialists in early palliative care models, as well as the impact of China’s unique population characteristics and different regional cultural customs on palliative care. Therefore, in our endeavor to promote and develop a palliative care model tailored to the Chinese context, it is crucial to address the following aspects. In order to maintain the integrity and organization of the palliative care system, it is imperative to establish a comprehensive framework consisting of co-built wards, palliative communities, palliative homes, and hospices (29). Special emphasis should be placed on providing comprehensive support to patients and their families, ensuring their comfort, and offering ongoing assistance to survivors in their mourning process (29). Additionally, it is crucial to consider the influence of China’s distinct population characteristics and cultural customs across various regions on the implementation of palliative care. Consequently, developing an appropriate palliative care pattern for Chinese circumstances is urgently needed.

E-warm pattern interventions are interdisciplinary palliative care technologies considering Chinese culture and circumstances (30). The E-warm model is defined as Early, Whole, Assessment, Re-evaluation, and MDT Management by acknowledging and incorporating local culture and traditions into practices (30). By implementing the E-warm model, our primary objective is to establish a comprehensive palliative care system and procedure that embodies Chinese attributes, encompassing diverse professional teams and intervention cycles, in order to deliver comprehensive, holistic, family-centered, and continuous care to patients and their families. Herein, we investigated the effectiveness of the combined early palliative care for NSCLC patients on the QoL, psychological well-being, pain, and nutrition state.

## Methods

### Study design and patients

An open, randomized, controlled trial was conducted from October 7, 2019, to October 25, 2021, in newly diagnosed NSCLC patients at the Chongqing University Cancer Hospital in Chongqing, China. The inclusion criteria considered inpatients and outpatient, and patients could be followed up in an outpatient or inpatient setting after being recruited. The study was open to patients who had Stage IIIB to IV advanced NSCLC within eight weeks of enrollment, treatment naïve or have not received disease-directed treatments, were 18 years or older, had a baseline Eastern Cooperative Oncology Group (ECOG) from 0 to 2, had expected survival time at least 24 weeks and had sufficient reading and cognitive skills. Patients who have already received palliative care services were not eligible.

The Chongqing University University Cancer Hospital’s Ethics Committee approved this study (CZLS2019177), registered at http://www.chictr.org.cn/ (ChiCTR2200062617). The study design was not revised after it began.

### Procedures

A flowchart of the combined early palliative care (CEPC) team process is shown in **Supplementary Figure S1**. The medical oncologists made medical decisions following the NCCN Guidelines and patient preferences (31). The E-warm model, which encompasses the principles of Early, Whole, Assessment, Re-evaluation, and MDT Management, is proposed (30). The term “Early” denotes the importance of early intervention, particularly in the context of advanced tumor patients, where early palliative care should be integrated into their anti-cancer treatment. The concept of “Whole” emphasizes the need for palliative care to be integrated throughout the entire cancer treatment process. “Evaluation” highlights the significance of dynamic assessment, allowing for continuous improvement of intervention strategies based on clinical feedback. Lastly, “MDT Management” underscores the necessity of Multi-Disciplinary Treatment being consistently applied throughout cancer treatment. In order to maintain the integrity and organization of the palliative care system, it is imperative to establish a comprehensive framework consisting of co-built wards, palliative communities, palliative homes, and hospices. Special emphasis should be placed on providing comprehensive support to patients and their families, ensuring their comfort, and offering ongoing assistance to survivors in their mourning process. Additionally, it is crucial to consider the influence of China’s distinct population characteristics and cultural customs across various regions on the implementation of palliative care. Currently, the E-warm model represents an initial palliative care approach that aligns with the aforementioned Chinese cultural customs and characteristics. The E-warm model focuses on establishing a palliative care system and procedure that incorporates Chinese characteristics, encompassing diverse professional teams and intervention cycles, in order to provide patients and their families with comprehensive, holistic, family-centered, and continuous care. For further details, please refer to the **Supplementary Appendix**.

A 1:1 randomization without stratification was used to assign eligible patients to either of the two groups within eight weeks of diagnosis. Professionals working for Palliative Care Services provided support and care for inpatients and outpatients. For 24 weeks, beginning within the first week of enrollment and continuing every month, patients met with a medical oncologist, an oncology nurse specialist, a dietitian, and a psychologist in the CEPC group. It was up to the patient, oncologist, or CEPC team to schedule additional palliative care visits. Randomly assigned SC patients did not have nutrition, pain, or psychology assessments except because of patient or oncologist requests. A single patient from the SC group did not cross over to the CEPC group if they received nutritional, psychological, or cancer pain consultations. Oncologic care was routinely provided to all study participants.

The early palliative care in the CEPC group focused on four basic elements: QoL, nutrition level, pain management, and psychological support (1). The QoL was evaluated by oncologists using the FACT-L scale, including the lung-cancer subscale (LCS) and Trial Outcome Index (TOI), which appraise the multidimensionality of the health-related QoL (function and symptom) (30) (2). Besides PG-SGA, dietitians assessed each patient’s nutritional intake, physical exams, and hematology tests. A nutritional intervention was initiated following the assessment results (3). Oncologists used an NRS to assess pain, and pain treatment was provided to the patient when necessary (4). The HADS, which evaluates anxiety (HADS-A) and depression (HADS-D) symptoms, and the Patient Health Questionnaire-9 (PHQ-9) were used by psychologists for psychological evaluations (8, 32). Psychologists provided psychotherapy to each patient and administered psychotropic medications when necessary.

The primary outcome was overall survival (OS). Secondary outcomes included changes in the QoL, nutrition state, pain, and psychological functioning. After enrollment, both groups were assessed every four weeks for QoL, nutritional level, pain status, and psychological factors. During CEPC weekly meetings, members discussed trial-related issues and potential solutions to improve the process to ensure all patients received coordinated interventions.

### Statistical analysis

SPSS 22.0 was used to analyze the data. Descriptive statistics were used to estimate frequency distributions, means, and standard deviations. For categorical variables, Fisher’s exact and χ^2^ tests were used to assess differences between groups for baseline characteristics and clinical outcomes. Independent-sample Student’s t-tests were used for continuous variables. Kaplan-Meier plots and log-rank tests were performed to determine whether combining early palliative care with other forms of treatment led to better OS. Data were analyzed based on the intention-to-treat method. Patients were randomly assigned, including those who died or were not followed up. An intention-to-treat analysis was performed using the last observation as the outcome. A per-protocol analysis of participants who fulfilled the protocol’s eligibility requirements was performed (see **Supplementary Table S1**). Statistical significance was defined as *p* < 0.05.

## Results

**Figure 1:**
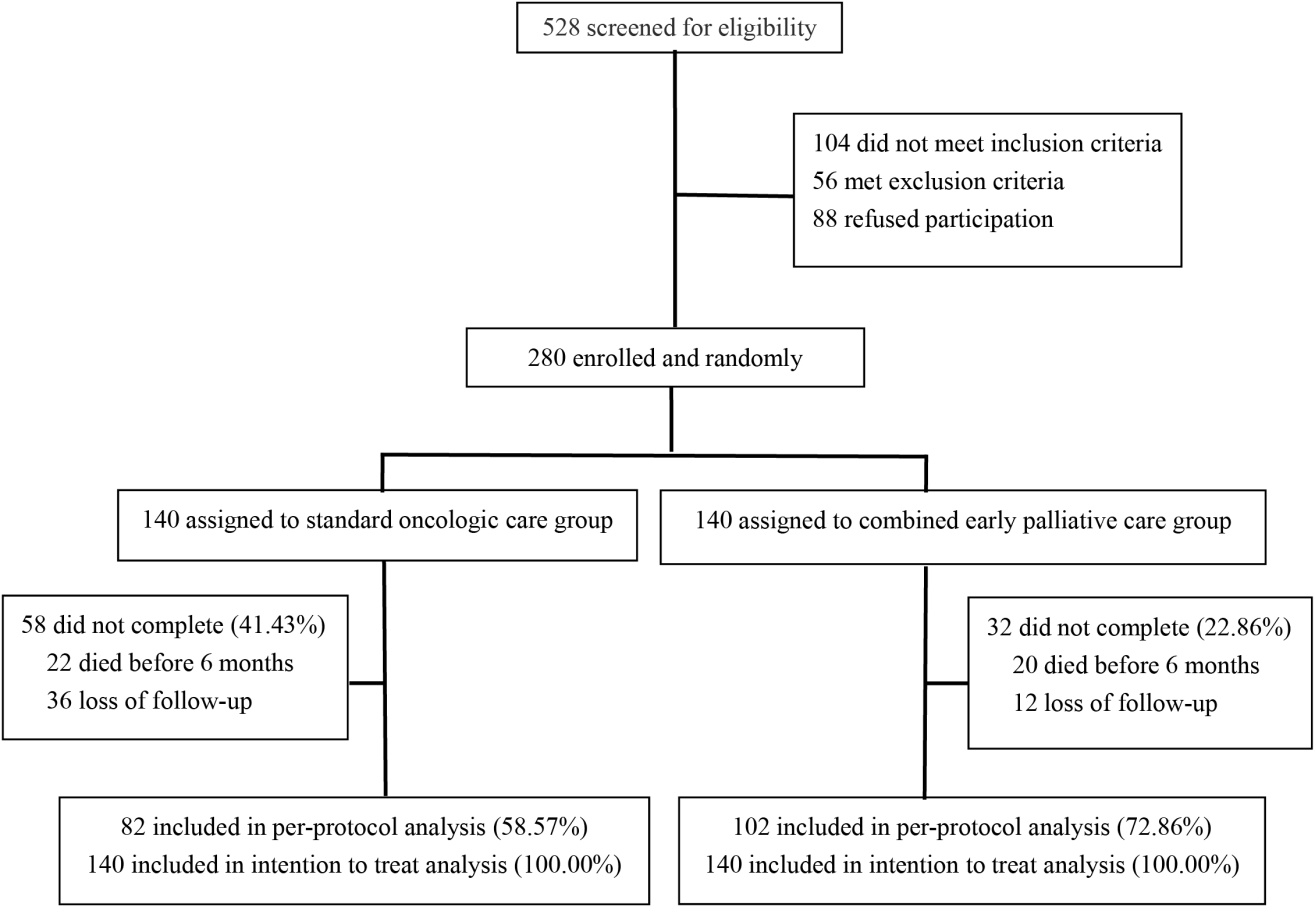
Trial profile.

A total of 528 NSCLC patients were assessed for participation. Finally, 280 were enrolled and randomly (1:1) assigned to CEPC or standard oncologic care (**Figure 1**). Ultimately, all 280 patients were included in the intention-to-treat analysis, and 184 patients were analyzed per protocol, 102 in the CEPC group and 82 in the SC group. The compliance at 24 weeks was 72.86 and 58.57% in the CEPC and SC groups. Both groups had similar demographics and baseline clinical characteristics (**Table 1**). Between the SC and CEPC groups, 24.29% (34/140) *vs*. 60.71% (85/140) received nutritional consultations, 20.00% (28/140) *vs*. 22.14% (31/140) received psychological consultations, and 27.14% (38/140) *vs*. 40.00% (56/140) received pain medications over the 24 weeks. Additionally, 41.43% (58/140) of patients in the SC group and 22.86% (32/140) in the CEPC group were no longer being followed up or died at the cutoff date (April 25, 2022).

**Table 1:** Patients’ Demographic and Baseline Characteristics.

Characteristic	Standard Care (n=140)	Combined Early Palliative Care (n=140)	*t/*χ^2^ */Z*	*P*
**Age, years**	62.9±10.36	62.02±10.36	0.71	0.478
**Sex—no.(%)**			0.16	0.690
Male	99 (70.71 %)	102 (72.86%)		
Female	41 (29.29%)	38 (27.14%)		
**Height,cm**	162.3±7.27	161.94±7.61	0.40	0.693
**Weight, kg**	59.26±9.52	59.09±10.21	0.14	0.887
**BMI, kg/m 2**	22.48±2.98	22.5±3.35	-0.06	0.954
**Waist, cm**	83.23±8.01	82.77±8.66	0.41	0.684
**ECOG—no.(%)**			1.77	0.412
0	20 (14.29%)	16 (11.43%)		
1	71 (50.71%)	82 (58.57%)		
2	49 (35.00%)	42 (30.00%)		
**Smoking status—no.(%)**			0.02	0.900
Former	93 (66.4%)	92 (65.71%)		
Never	47 (33.57%)	48 (34.29%)		
**Histology—no.(%)**			0.81	0.668
Adenocarcinoma	91 (65.00%)	97 (69.29%)		
Squamous cell	47 (33.57%)	42 (30.00%)		
Other	2 (1.43%)	1 (0.71%)		
**AJCC cancer stage—no.(%)**			0.52	0.771
IIIB	15 (10.71%)	18 (12.86%)		
IIIC	14 (10.00%)	16 (11.43%)		
IV	111 (79.29%)	106 (75.71%)		
**PG-SGA score—no.(%)**			0.02	0.990
0–1	19 (13.57%)	19 (13.57%)		
2–8	87 (62.14%)	86 (61.43%)		
≥9	34 (24.29%)	35 (25.00%)		
**NRS score—no.(%)**			0.27	0.874
No pain (0)	67 (47.86%)	65 (46.33%)		
Mild pain (1-3)	55 (39.29%)	54 (38.57%)		
Moderate pain (4–6)	18 (12.86%)	21 (15.00%)		
Severe pain (7-10)	0	0		
**Assessment of mood symptoms**				
**HADS**				
Anxiety subscale (HADS-A)	3.54±2.73	3.91±3.09	-0.32	0.746
Depression subscale (HADS-D)	4.41±3.51	4.16±3.28	-1.272	0.203
**PHQ-9** Depression severity			2.18	0.535
No (0-4)	93 (66.43%)	89 (63.57%)		
Mild (5-9)	38 (27.14%)	46 (32.86%)		
Moderate(10-14)	8 (5.71%)	4 (2.86%)		
Moderately severe(15-19)	1(0.71%)	1(0.71%)		
**Scores on quality-of-life measures**				
FACT-L scale	106.14±12.66	105.94±12.16	0.14	0.892
Lung-cancer subscale	27.84±4.14	28.08±3.67	-0.51	0.612
Trial Outcome Index	67.18±9.53	67.51±8.54	-0.30	0.762

Data are means ±SD or n (%). Percentages might not total 100% because of rounding. Abbreviations: ECOG, Eastern Cooperative Oncology Group; AJCC, American Joint Committee on Cancer; PG-SGA, Patient-Generated Subjective Global Assessment; NRS, Numerical Rating Scale; HADS, Hospital Anxiety and Depression Scale; PHQ-9, Patient Health Questionnaire-9; FACT-L, Functional Assessment of Cancer Therapy-Lung.

### Baseline characteristics

The baseline characteristics were similar for both groups (**Table 1**). Patients were matched on age, sex, height, weight, BMI, ECOG, smoking state, histology, and neoplasm staging. The groups did not differ regarding the baseline nutritional assessment, pain evaluation, emotional symptoms, or QoL (**Table 1**).

### Key characteristics at baseline and 24 weeks

In the SC group, the baseline nutrition assessment and pain level did not significantly differ at baseline and after 24 weeks (**Table 2**). In contrast, the level of PG-SGA, NRS, HADS, PHQ-9, and QoL of the CEPC group markedly improved after 24 weeks (*p* < 0.05, **Table 2**). Subsequent analysis of participants who completed the 24-week intervention revealed no statistical differences in factors such as age, gender, and cancer stage between the SC group and CEPC group (see **Supplementary Table S1**).

**Table 2:** Patients’ key characteristics at baseline and 24 weeks by intention-to-treat analysis.

Characteristic	Standard Care		*P*	Combined Early Palliative Care		*P*
	Baseline (n=140)	6 months (n=140)		Baseline(n=140)	6 months (n=140)	
**PG-SGA score—no.(%)**			0.221			<0.001
0–1	19 (13.57%)	30 (21.43%)		19 (13.57%)	53 (37.86%)	
2–8	87 (62.14%)	78 (55.71%)		86 (61.43%)	67 (47.86%)	
≥9	34 (24.29%)	32 (22.86%)		35 (25.00%)	20 (14.29%)	
**NRS score—no.(%)**			0.396			<0.001
No pain (0)	67 (47.86%)	76 (54.29%)		65 (46.33%)	103 (73.57%)	
Mild pain (1-3)	55 (39.29%)	52 (37.14%)		54 (38.57%)	29 (20.71%)	
Moderate pain (4–6)	18 (12.86%)	12 (8.57%)		21 (15.00%)	8 (5.71%)	
Severe pain (7-10)	0	0		0	0	
**Assessment of mood symptoms**						
**HADS**						
Anxiety subscale (HADS-A)	3.54 ± 2.73	2.66 ± 2.86	0.009	3.91 ± 3.09	1.45 ± 2.86	<0.001
Depression subscale (HADS-D)	4.41 ± 3.51	3.54 ± 4.61	0.074	4.16 ± 3.28	1.5 ± 2.05	<0.001
**PHQ-9 major depressive syndrome**			0.011			<0.001
Depression severity	93 (66.43%)	111 (79.29%)		89 (63.57%)	127 (90.71%)	
No (0-4)	38 (27.14%)	20 (14.29%)		46 (32.86%)	12 (8.57%)	
Mild (5-9)	8 (5.71%)	4 (2.86%)		4 (2.86%)	1 (0.71%)	
Severe pain (7-10)	1(0.71%)	5 (3.57%)		1(0.71%)	0	
**Scores on quality-of-life measures**						
FACT-L scale	106.14 ± 12.66	111.66 ± 14.9	0.001	105.94 ± 12.16	117.81 ± 11.15	<0.001
Lung-cancer subscale	27.84 ± 4.14	29.64 ± 3.94	<0.001	28.08 ± 3.67	30.9 ± 2.96	<0.001
Trial Outcome Index	67.18 ± 9.53	70.66 ± 11.35	0.006	67.51 ± 8.54	75.62 ± 8.62	<0.001

Data are means ± standard deviations (SD) or n (%). Percentages might not total 100% because of rounding. This test was conducted with by intention-to-treat analysis.

Data are means ± SD or n (%). Percentages might not total 100% because of rounding. PG-SGA, Patient-Generated Subjective Global Assessment; NRS, Numerical Rating Scale; HADS, Hospital Anxiety and Depression Scale; PHQ-9, Patient Health Questionnaire-9; FACT-L, Functional Assessment of Cancer Therapy-Lung.

### Nutrition, pain, mood symptoms, health-related QoL, and survival analysis

CEPC and SC patients were assessed by PG-SGA at baseline, and after 24 weeks, all participants were re-assessed. Additionally, the CEPC group (14.29 *vs*. 22.86%, severe malnutrition; 47.86 *vs*. 55.71%, moderate or mild malnutrition; and 37.86 *vs*. 22.86%, no malnutrition) had a better nutrition level than the SC group (*p* = 0.007) (**Table 3**). Moreover, the CEPC group had a much lower NRS score than the SC group after 24 weeks (*p* = 0.003) (**Table 3**).

**Table 3:** Intention to treat analyses of patients’ characteristics at 24 weeks.

Characteristic	Standard Care (n=140)	Combined Early Palliative Care (n=140)	*t/*χ^2^ */Z*	*P*值
**Height,cm**	161.99 ± 7.31	161.89 ± 7.91	0.11	0.915
**Weight, kg**	60.02 ± 9.27	60.38 ± 10.79	-0.27	0.788
**BMI, kg/m 2**	22.83 ± 2.95	22.96 ± 3.55	-0.31	0.759
**Waist, cm**	82.01 ± 8.78	84.08 ± 7.23	-2.82	0.090
**PG-SGA score—no.(%)**			9.98	**0.007**
No malnutrition (0–1)	30 (21.43%)	53 (37.86%)		
Mild or moderate malnutrition (2–8)	78 (55.71%)	67 (47.86%)		
Severe malnutrition (≥9)	32 (22.86%)	20 (14.29%)		
**NRS score—no.(%)**			11.40	**0.003**
No pain (0)	76 (54.29%)	103 (73.57%)		
Mild pain (1-3)	52 (37.14%)	29 (20.71%)		
Moderate pain (4–6)	12 (8.57%)	8 (5.71%)		
Severe pain (7-10)	0	0		
Assessment of mood symptoms				
HADS				
Anxiety subscale (HADS-A)	2.66 ± 2.86	1.45 ± 2.86	4.13	**< 0.001**
Depression subscale (HADS-D)	3.54 ± 4.61	1.5 ± 2.05	4.77	**< 0.001**
**PHQ-9** Depression severity			9.88	**0.020**
No (0-4)	111 (79.29%)	127 (90.71%)		
Mild (5-9)	20 (14.29%)	12 (8.57%)		
Moderate(10-14)	4 (2.86%)	1 (0.71%)		
Moderately severe(15-19)	5 (3.57%)	0		
Scores on quality-of-life measures				
FACT-L scale	111.66 ± 14.90	117.81 ± 11.15	-3.907	**< 0.001**
Lung-cancer subscale	29.64 ± 3.94	30.90 ± 2.96	-3.017	**0.003**
Trial Outcome Index	70.66 ± 11.35	75.62 ± 8.62	-4.12	**< 0.001**

Data are means ± standard deviations (SD) or n (%). Percentages might not total 100% because of rounding. This test was conducted with by intention-to-treat analysis.

Data are means ± SD or n (%). Percentages might not total 100% because of rounding. PG-SGA, Patient-Generated Subjective Global Assessment; NRS, Numerical Rating Scale; HADS, Hospital Anxiety and Depression Scale; PHQ-9, Patient Health Questionnaire-9; FACT-L, Functional Assessment of Cancer Therapy-Lung.

Furthermore, the CEPC group had significantly lower levels of anxiety and depression at 24 weeks when assessed by both HADS and PHQ-9 (*p* < 0.05) than SC group patients. Nonetheless, the proportion of patients prescribed antidepressant drugs was similar (about 20%, *p* = 1.000). In the PHQ-9 depression severity test, the groups significantly differed, considering depression severity scores (*p* = 0.020). The per-protocol analyses showed similar results for HADS mood symptoms (**Supplementary Table S1**).

In the present study, a total of 15 patients (10.71%) in the SC group received palliative care consultations upon request, either by the patient or the oncologist, within the initial 24-week period. The primary purpose of these consultations was to address symptom management. Among these patients, 11 received a single visit, while 4 received two visits.

**Figure 2:**
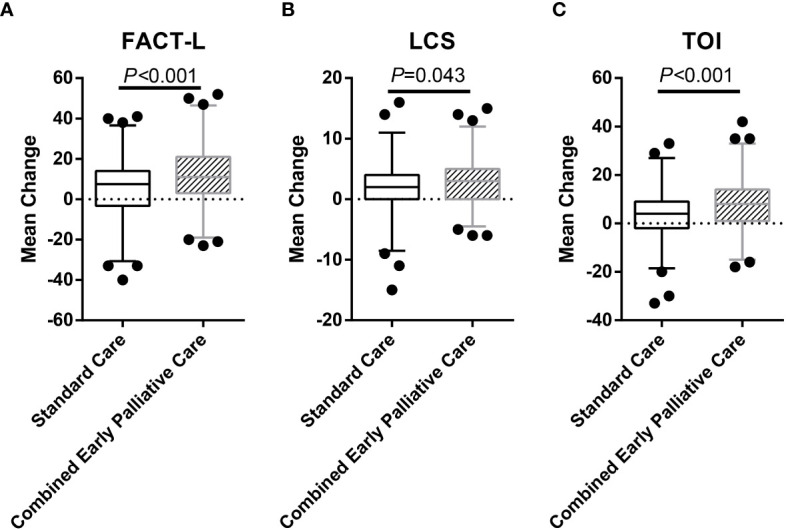
Mean Change in Quality-of-Life Scores from Baseline to 24 Weeks in the Two Study Groups. The study group was the independent variable, and the two-sided independent-sample Student’s t-tests showed a trend toward a significant between-group difference in the mean ( ± SD) change in scores from baseline to 24 weeks on the FACT-L scale (5.51 ± 14.04 in the SC group *vs*. 11.77 ± 14.89 in the CEPC group; differences between groups, 6.26; 95% confidence interval (CI), 2.83 to 9.68; *p* < 0.001) **(A)**. A significant between-group difference was detected in the mean change in scores on the LCS (1.80 ± 4.33 and 2.80 ± 3.81 in the two groups, respectively; the difference between groups, 0.99; 95% CI, 0.03 to 1.96; *p* = 0.043) **(B)**, as well as a significant between-group difference in the mean change in TOI scores(3.45 ± 10.41 *vs*. 8.03 ± 10.83; the difference between groups, 4.58; 95% CI, 2.07 to 7.09; *p* < 0.001) **(C)**. Data from 140 patients in the SC group and 140 patients in the CEPC group by intention-to-treat analysis. I bars indicate 95% confidence intervals.

**Figure 3:**
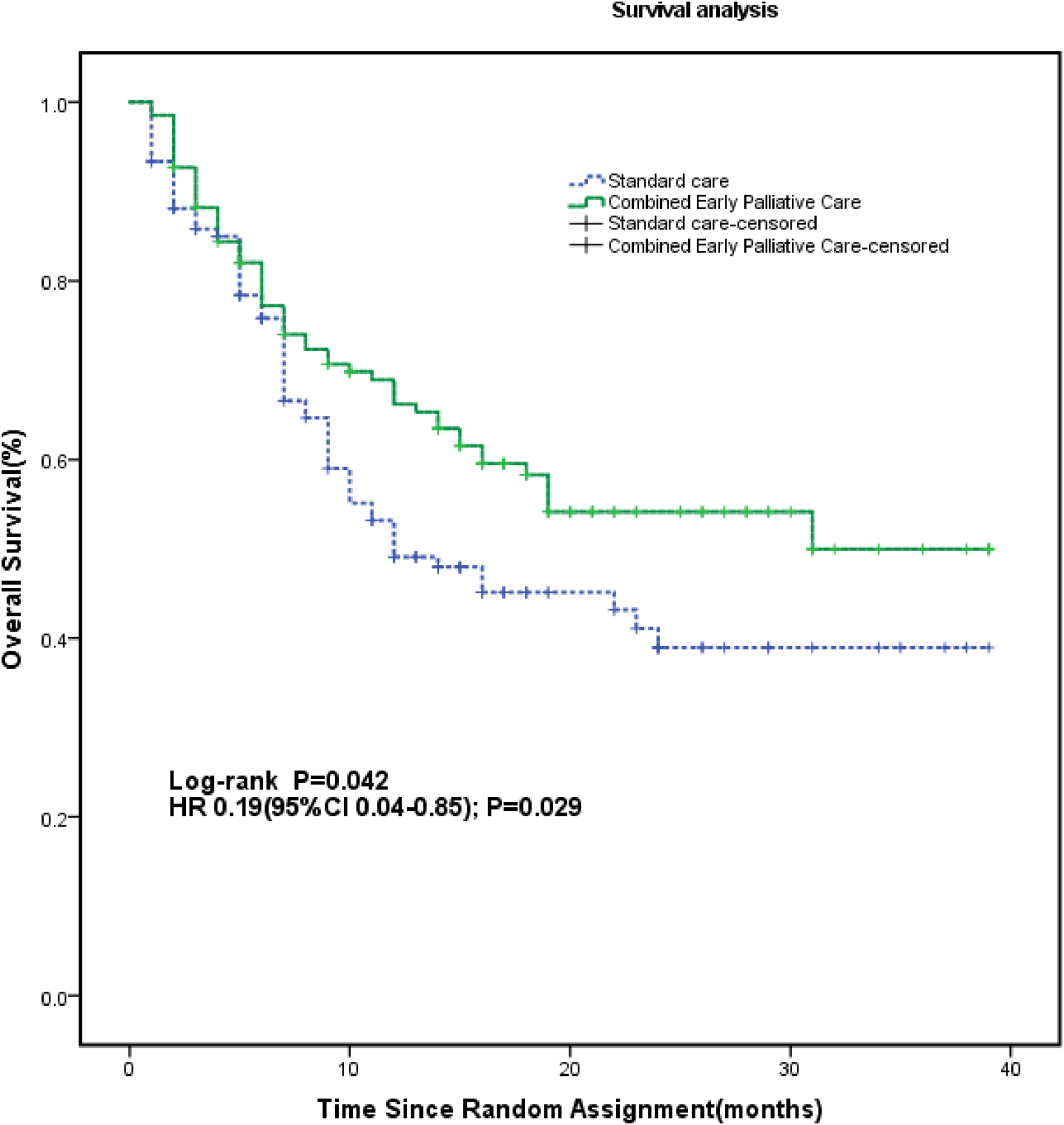
Kaplan-Meier Survival Estimates According to Study Group. The survival rate was calculated from enrollment through the time of death if it occurred during the study period or until January 1, 2023, when the data were censored. The median OS was 24.6 months (95% CI, 21.8 to 27.5) for patients assigned to the CEPC group (n = 140 patients) compared to 20.4 months (95% CI, 17.4 to 23.4) for patients in the SC group (n = 140 patients) (HR, 0.19; 95% CI, 0.04 to 0.85; *p* = 0.029). HR, hazard ratio.

The combined early palliative care treatment significantly affected the QoL, improving not only the FACT-L scale (117.81 ± 11.15 *vs*. 111.66 ± 14.90; *p* < 0.001) but also the LCS (30.90 ± 2.96 *vs*. 29.64 ± 3.94; *p* = 0.003) and TOI (75.62 ± 8.62 *vs*. 70.66 ± 11.35; *p* < 0.001) than the SC group after 24 weeks (**Table 3**). From baseline to the 24th week, CEPC patients increased their mean FACT-L score by 11.77 points and SC patients by only 5.51 points (*p* < 0.001) (**Figure 2**). CEPC patients had significantly better survival than SC patients (median OS, 24.6 *vs*. 20.4 months, *p* = 0.042; HR, 0.19; 95% CI, 0.04 to 0.85, *p* = 0.029, **Figure 3**).

## Discussion

This study examined the effects of combined early palliative care (according to the E-warm concept) for NSCLC patients. When palliative care was integrated early into standard oncology care, NSCLC patients presented a clinically meaningful improvement in QoL, nutritional state, pain management, psychological level, and survival benefit. NSCLC patients have shorter survival when depressed, a poor nutritional level, and lower QoL (31). Further, when Early Palliative Care is integrated with Standard Oncologic Care, anticancer therapy can be optimally and appropriately administered, especially in the final stages of the illness (8, 32, 33). By receiving early referrals to palliative care, patients might manage their symptoms better, improving their condition and having a longer life expectancy (9, 34). Nevertheless, future studies are required to confirm these hypotheses.

Considering the progressive nature of NSCLC, improving patients’ quality of life, nutrition level, pain management, and psychological status is a major challenge (7). Our previous study has shown that early palliative care improved patients’ QoL in the FACT-L (30), consistent with this study and previous reports on metastatic NSCL patients in the New England Journal of Medicine (10). Palliative care integration in oncology care has been shown to improve quality of life in three previous trials at 12 weeks (8, 35, 36), and in three other trials at later time points (37–39). At 24 weeks, the intervention proposed in our trial significantly improved quality of life.

As a result of our findings, early and planned palliative care consultations are critical for patients to discuss all aspects of palliative care at their own pace. These approach contrasts with the usual care group, in which palliative care consultations were only arranged on demand and often. In the era of rapid development of targeted therapy and immunotherapy, comparing patients assigned to CEPC and those assigned to SC is of great interest. The CEPC group had better survival benefits and QoL. Besides, the depression scores significantly differed between the two groups. Thus, this did not result from a difference in antidepressant use between the groups.

Previous studies have demonstrated the benefits of supportive care in terms of QoL, symptom control, and mood management (30, 40, 41). Similar to previous studies (8, 38), we observed an improvement in overall survival. Despite the lack of a clear mechanism behind a better overall survival in this setting, more aggressive early palliative care treatment regimens have been suggested as an explanation (8, 38). There is evidence that the improved nutritional and psychological status of the CEPC group may contribute to the prolonged OS. Anxiety and depression have been linked to a significant increase in cancer-specific mortality (42). Furthermore, individualized nutritional interventions have been shown to reduce 30-day mortality (43). Impacts of nutrition and psychological status may play a significant role in immune response (41, 44–46), which improves long-term prognosis. There have, however, been few studies specifically investigating the effectiveness of multiple support systems in cancer patients.

In a recent study, nutrition and psychology interdisciplinary palliative care enhanced the OS in advanced esophagogastric cancer patients by alleviating symptoms (32). Our findings are similar to those from other researchers in which CEPC improved the QoL of advanced cancer patients (35, 47, 48). Our intervention might have had an effect on aggressive treatment choices of patients because the role of the effect of combined early palliative care group in our trial was large.

This randomized controlled trial has several advantages. First, palliative care interventions were provided for 24 weeks, and many studies have shorter intervention times (8, 35, 47, 49). Furthermore, no model of palliative care is suitable for Chinese conditions. Moreover, our study provided a Chinese-oriented model for palliative care.

However, our current study also has some limitations. First, it was conducted at a single institution, with only Chinese patients. Thus, its generalizability to people from different races and settings might be limited. An additional optimization is needed to customize this palliative care model to meet the needs of different cultures and resources. Second, a potential bias was introduced because participants and investigators were not masked in the group assignment. Finally, the intention-to-treat analysis is conservative if all missing data are carried forward from the last observation. This would suggest that the treatment effect of combined early palliative care might be greater than what we reported here.

In summary, we examined combined early palliative care (based on the E-warm concept) among NSCLC patients. Early palliative care may benefit survival rates, quality of life, psychological well-being, pain management, and nutrition. It would be beneficial to optimize and standardize further.

## Data availability statement

The original contributions presented in the study are included in the article/**Supplementary Material**. Further inquiries can be directed to the corresponding author.

## Ethics statement

The Ethics Committee of the Chongqing University Cancer Hospital approved this study (CZLS201917). Informed consent was obtained from all patients before they participated in the study.

## Author contributions

MC and HuY initiated the project, designed and performed experiments, analysed the data and drafted the manuscript. LY, HoY, HC, LL, LM, SL, LT, SW enrolled and followed up the patients. HuY and MC designed the project, obtained funding, helped with the writing of the paper, and finalized the manuscript. All authors contributed to the article and approved the submitted version.
