# Supplementary figures and images for: Combined early palliative care for non-small-cell lung cancer patients: a randomized controlled trial in Chongqing, China
Source: Front Oncol. 2023 Sep 14;13:1184961. doi: 10.3389/fonc.2023.1184961 (PMC10539600; doi:10.3389/fonc.2023.1184961)

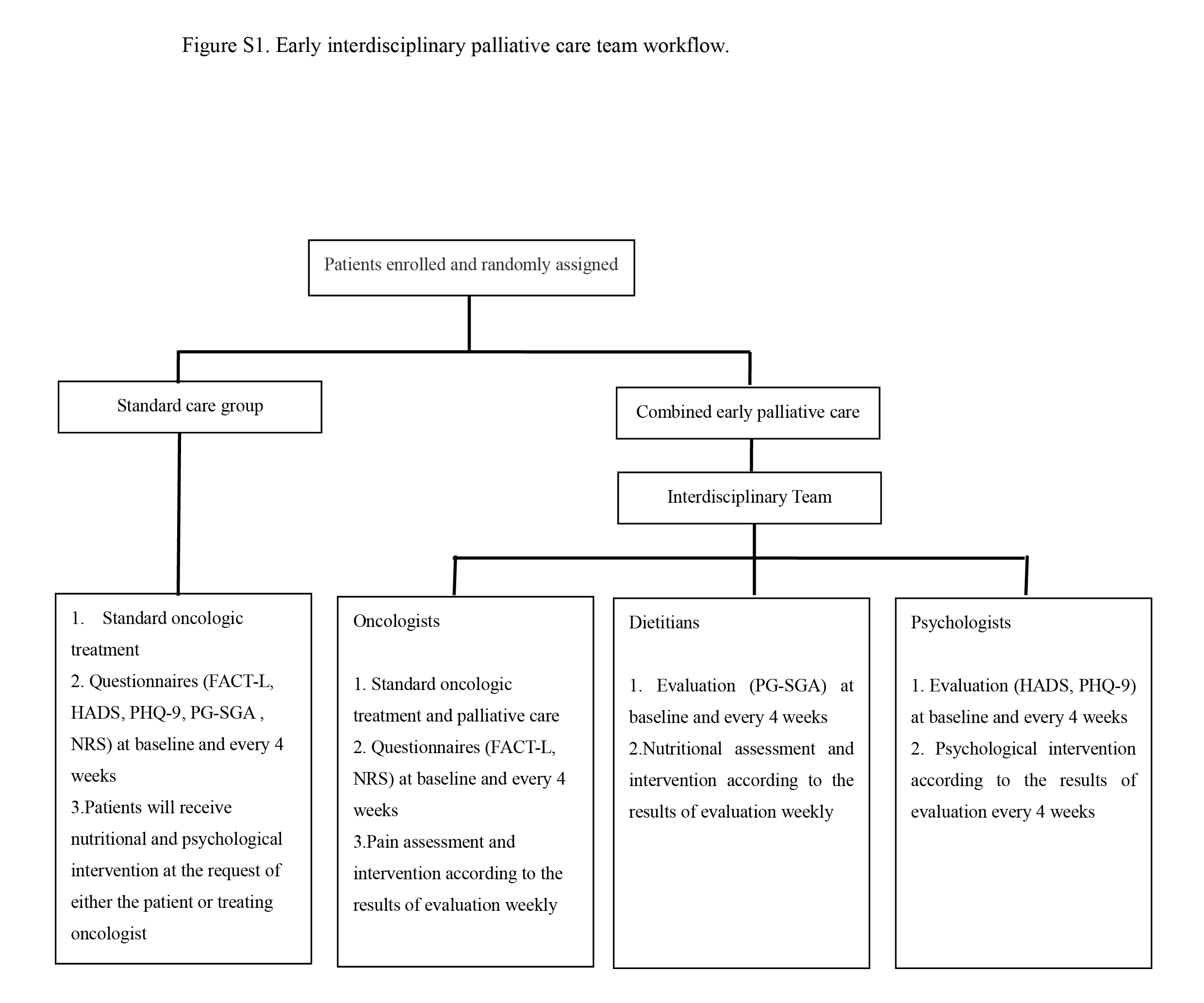

Supplement: Supplementary file 1 [file Image_1.tif]
