# Supplementary material for: Combined early palliative care for non-small-cell lung cancer patients: a randomized controlled trial in Chongqing, China
Source: Front Oncol. 2023 Sep 14;13:1184961. doi: 10.3389/fonc.2023.1184961 (PMC10539600; doi:10.3389/fonc.2023.1184961)
Supplement: Supplementary file 2 [file Table_1.doc]

**Table S1: Per-Protocol analyses of Patients’ Characteristics at 24 Weeks.**

| **Characteristic** | **Standard Care (n=82)** | **Combined Early Palliative Care (n=102)** | ***t/*χ^2^*/Z*** | ***P*** |
| --- | --- | --- | --- | --- |
| **Age, years** | 63.83±11.11 | 62.64±10.55 | 0.74 | 0.458 |
| **Sex—no.(%)** |  |  | 0.36 | 0.547 |
| Male | 57 (69.51 %) | 75 (73.53%) |  |  |
| Female | 25 (30.49%) | 27 (26.47%) |  |  |
| **Height,cm** | 161.41±7.95 | 161.45±8.26 | -0.03 | 0.974 |
| **Weight, kg** | 59.83±9.73 | 60.92±10.69 | -0.71 | 0.478 |
| **BMI, kg/m 2** | 22.90±3.10 | 23.30±3.35 | -0.83 | 0.410 |
| **Waist, cm** | 82.02±7.54 | 84.33± 6.97 | -1.75 | 0.082 |
| **AJCC cancer stage—no.(%)** |  |  | 0.27 | 0.875 |
| IIIB | 9 (10.98%) | 13 (12.75%) |  |  |
| IIIC | 10 (12.20%) | 14 (13.73%) |  |  |
| IV | 63 (76.83%) | 75 (73.53%) |  |  |
| **PG-SGA score—no.(%)** |  |  | 1.88 | 0.390 |
| No malnutrition (0–1) | 24 (29.27 %) | 31 (30.39%) |  |  |
| Mild or moderate malnutrition (2–8) | 51 (62.20%) | 56 (54.90%) |  |  |
| Severe malnutrition (≥9) | 7 (8.54 %) | 15 (14.71%) |  |  |
| **NRS score—no.(%)** |  |  | 5.96 | 0.051 |
| No pain (0) | 47(57.32%) | 75 (73.53%) |  |  |
| Mild pain (1-3) | 31(37.80%) | 22 (21.57%) |  |  |
| Moderate pain (4–6) | 4 (4.88%) | 5 (5.90%) |  |  |
| Severe pain (7-10) | 0 | 0 |  |  |
| **Assessment of mood symptoms** |  |  |  |  |
| **HADS** |  |  |  |  |
| Anxiety subscale (HADS-A) | 2.17±2.56 | 1.34±2.56 | 2.77 | 0.006 |
| Depression subscale (HADS-D) | 2.19±2.99 | 1.42±1.70 | 2.20 | 0.029 |
| **PHQ-9**  Depression severity |  |  | 1.31 | 0.519 |
| No (0-4) | 73 (89.02%) | 93 (91.18%) |  |  |
| Mild (5-9) | 8 (9.56%) | 9 (8.82%) |  |  |
| Moderate(10-14) | 1 (1.22 %) | 0 |  |  |
| **Scores on quality-of-life measures** |  |  |  |  |
| FACT-L scale | 116.88±11.43 | 118.08±9.81 | -0.77 | 0.444 |
| Lung-cancer subscale | 30.96±2.83 | 30.86±2.98 | 0.23 | 0.820 |
| Trial Outcome Index | 74.98±7.81 | 75.77±7.52 | -0.70 | 0.487 |

Data are means ±SD or n (%). Percentages might not total 100% because of rounding. This test was conducted with by per-protocol analysis. Abbreviations: PG-SGA, Patient-Generated Subjective Global Assessment; NRS, Numerical Rating Scale; HADS, Hospital Anxiety and Depression Scale; PHQ-9, Patient Health Questionnaire-9; FACT-L, Functional Assessment of Cancer Therapy-Lung.
