# Supplementary material for: Combined early palliative care for non-small-cell lung cancer patients: a randomized controlled trial in Chongqing, China
Source: Front Oncol. 2023 Sep 14;13:1184961. doi: 10.3389/fonc.2023.1184961 (PMC10539600; doi:10.3389/fonc.2023.1184961)
Supplement: Supplementary file 3 [file DataSheet_1.pdf]

Supplementary Appendix

Supplemental Methods

E-warm pattern interventions are interdisciplinary palliative care technologies considering Chinese culture and circumstances [1]. The term "Early" denotes the importance of early intervention, particularly in the context of advanced tumor patients, where early palliative care should be integrated into their anti-cancer treatment. The concept of "Whole" emphasizes the need for palliative care to be integrated throughout the entire cancer treatment process. "Evaluation" highlights the significance of dynamic assessment, allowing for continuous improvement of intervention strategies based on clinical feedback. Lastly, "MDT Management" underscores the necessity of Multi-Disciplinary Treatment being consistently applied throughout cancer treatment.

We recruited patients with advanced cancer from the Geriatric Oncology, Medical Oncology, and Thoracic Oncology departments of Chongqing University Cancer Hospital. Patients were identified for recruitment by a trained clinical research assistant (who attend weekly staff meetings) and the treating oncologists. Both outpatients and inpatients were considered for inclusion. Study participants will be randomized in a 1:1 fashion to the combined early palliative care (CEPC) or standard oncological care (SC) intervention. Professionals working for Palliative Care Services provided support and care for inpatients and outpatients.

Participants who are randomized to the CEPC group will meet with the CEPC group within one week of providing informed consent. For 24 weeks, beginning within the first week of enrollment and continuing every month, patients met with a medical oncologist, an oncology nurse specialist, a dietitian, and a psychologist in the CEPC group. It was up to the patient, oncologist, or CEPC team to schedule additional palliative care visits. Randomly assigned SC patients did not have nutrition, pain, or psychology assessments except because of patient or oncologist requests. A single patient from the SC group did not cross over to the CEPC group if they received nutritional, psychological, or cancer pain consultations. Oncologic care was routinely provided to all study participants.

The E-warm combined early palliative care intervention model is outlined in 1.1 and in the accompanying Appendix A.

1.1. E-warm model combined early palliative care intervention

Palliative care is comprehensive, coordinated interdisciplinary care for patients and

families facing a potentially life-threatening illness. The Palliative Care Services (PCS) consists of specially trained teams of professionals that provide care and support in inpatient and outpatient settings. The PCS is comprised of medical oncologists, oncology nurse specialists, palliative care physicians, palliative care nurses, social workers, religious workers, bereavement specials, and volunteers. While the specific assistance and support provided by the PCS depends on individual patient and family needs and preferences, it may include:

1. pain and symptom management,
2. psychosocial and spiritual support,
3. nutritional treatment for malnutrition,
3. assistance with treatment choices,
4. help in planning for care in the community,
5. bereavement support and referral.

1.1.1. Combined early palliative care intervention

Patients in the CEPC group of the study will be required to meet with the CEPC group within one weeks of enrolling in the study. An attending physician/nurse practitioner from the CEPC will perform the initial assessment. We have developed general guidelines to ensure a uniform and reproducible intervention between the participating hospitals and palliative care providers. The clinicians will use the included guidelines – Palliative Care Intervention (Appendix A) as a tool to provide comprehensive symptom management and psychosocial, spiritual, and emotional support to patients and their families. We will also require the palliative care to document how much of the visit was spent on each element of the intervention guideline.

The initial assessment will occur in the outpatient clinic, unless the patient is admitted to the hospital prior to the assessment. If study participants are admitted to the hospital, the CEPC group will perform an inpatient consult. Once patients are discharged from the hospital, they will be followed outpatients pursuant to the study guidelines.

The CEPC group will follow each patient on a regular basis in conjunction with his or her primary oncologist. To avoid requiring more frequent clinic visits, the CEPC group will make an effort to meet with patients at their regularly scheduled visits, such as appointments with their oncologists and chemotherapy or radiation sessions. The CEPC group will make every attempt to see patients each time they come into the cancer hospital. However, patients will meet with the CEPC group a minimum of every four weeks. Study patients and their family will arrange additional visits at their discretion. If study participants appear distress as a result of the required

questionnaires, the palliative care team will be available to provide counseling.

The clinicians will review patients on the combined early palliative care group at a weekly thoracic oncology meeting. The CEPC group and the treating oncologist will discuss active patient issues to ensure a team approach to the patient's care. If the weekly thoracic oncology meeting does not take place, the CEPC group and the treating oncologist will communicate via online video conference. The CEPC group and treating oncologist will discuss major changes in patients' status or urgent issues on a timely via online video conference or telephone.

1.2. Standard oncological care intervention

Patients randomized to the standard oncological care group will be seen by the palliative care team at the request of either their treating oncologist or the patients or their families. We will not deny participants the opportunity to meet with the palliative care team at any time. When participants on the standard group meet with the palliative care team, their care will not be standardized. The study anticipates that the palliative care team will follow and treat these patients as they would any other cancer patient receiving palliative care. Participants on this group who experience distress related to completing the questionnaires will be referred to their primary oncologist for counseling.

1.3. Data Collection

Training sessions by oncologists to inform palliative care nurses and the physician about cancer treatments typically administered early in the disease trajectory, and their associated side-effects. These training sessions were provided before the initiation of the trial and deemed necessary because the palliative care team had little experience of providing early palliative care. One training session included the use of the intervention documents and standard training for the study protocol.

The types of patient and caregiver surveys and the frequency of their administration are detailed below. As described, we will assess QOL by the FACT-Lung (FACT-L) [2], mood by the HADS and Patient Health Questionnaire (PHQ-9) [3,4], and nutritional assessment by the PG-SGA at baseline and at 4, 8, 12, 16, 20, and 24 weeks. Enrolled patients completed follow-up questionnaires administered by the research nurse and 4-weekly thereafter until death. The research nurse will use a number of methods to administer questionnaires in order to accommodate patients' individual response preferences (in person or telephonic) and our desire to have them completed as close as possible to the scheduled assessment times. If patients do not have a scheduled visit at an assessment time and would prefer not to do a telephonic questionnaire, the questionnaire will be mailed to their home with a stamped,

addressed envelope.

Appendix A: Palliative Care Guidelines

Illness understanding/education<br>Inquire about illness and prognostic understanding<br>Offer clarification of treatment goals
Symptom management – Inquire about uncontrolled symptoms with a focus on:<br>Pain<br>Pulmonary symptoms (cough, dyspnea)<br>Fatigue and sleep disturbance<br>Mood (depression and anxiety)<br>Malnutrition<br>Gastrointestinal (anorexia and weight loss, nausea and vomiting, constipation)
Decision-making<br>Inquire about mode of decision-making<br>Assist with treatment decision-making, if necessary
Faith and religion<br>Based on Chinese traditional culture support
Coping with life threatening illness<br>Patient<br>Family/family caregivers
Referrals/Prescriptions<br>Identify care plan for future appointments<br>Indicate referrals to other care providers<br>Note new medications prescribed

References

1.Chen M, Yang L, Yu H, et al. Early Palliative Care in Patients With Non-Small-Cell Lung Cancer: A Randomized Controlled Trial in Southwest China. Am J Hosp Palliat Care, 2022;39:1304-1311.
2.Chen M, Ma L, Yu H, et al. JK5G postbiotics attenuate immune-related adverse events in NSCLC patients by regulating gut microbiota: a randomized controlled trial in China. Front. Oncol, 2022; 13:1155592.
3.Temel JS, Greer JA, Muzikansky A, et al. Early palliative care for patients with metastatic non-small-cell lung cancer. N Engl J Med, 2010;363:733-742.
4.Lu Z, Fang Y, Liu C, et al. Early Interdisciplinary Supportive Care in Patients With Previously Untreated Metastatic Esophagogastric Cancer: A Phase III Randomized Controlled Trial. J Clin Oncol, 2021;39:748-756.
